# Differential Responses and Temporal Lags of Heterotrophic and Autotrophic Respiration to Plant Activity in a Forest Ecosystem

**DOI:** 10.3390/plants15081175

**Published:** 2026-04-10

**Authors:** Dongmin Seo, Minyoung Lee, YoungSang Lee, Jeaseok Lee

**Affiliations:** 1 Department of Biological Science, Konkuk University, Seoul 05029, Republic of Korea; adrea5957@konkuk.ac.kr (D.S.); my991004@naver.com (M.L.); 2 Ecosystem Change Research Team, National Institute of Ecology, Seocheon 33657, Republic of Korea; lys890309@nie.re.kr

**Keywords:** soil respiration, heterotrophic respiration, autotrophic respiration, sap flux density, photosynthesis, time lag

## Abstract

Assimilated carbon allocation to belowground processes may influence soil respiration (Rs). Because Rs includes autotrophic respiration (Ra) and heterotrophic respiration (Rh), different root and microbial responses complicate the separation of these effects. In a temperate deciduous broadleaf forest, we used sap flux density and estimated photosynthesis as indicators of plant activity. Total soil respiration and heterotrophic respiration were measured using automated chambers, and autotrophic respiration was estimated as Rs minus Rh. We examined the overall responses and time lags of respiration components. Ra showed positive relationships with sap flux density and estimated photosynthesis (*R*^2^ = 0.37 and 0.30, *p* < 0.05), whereas Rh showed weaker relationships (*R*^2^ = 0.20 and 0.15, *p* < 0.05). In lagged cross-correlation analyses using high-resolution data, Rs and Ra showed maximum responses 13 h after plant activity changes, whereas Rh showed no lag response (*p* > 0.05). These results suggest that associations with plant activity were clearer for Ra than Rh, and that the detected lagged response of soil respiration was more consistent with partitioned Ra than Rh. However, because Ra was estimated as Rs minus Rh, these patterns should be interpreted cautiously. Considering the responses and time lags of respiration components may improve ecosystem carbon cycling predictions.

## 1. Introduction

Increases in atmospheric carbon dioxide (CO_2_) contribute to climate warming and alter ecosystem structure and function [1,2]. These changes modify plant physiological activity in forest ecosystems, thereby influencing carbon uptake through photosynthesis and carbon release through soil and plant respiration [3]. If the net carbon sink strength of forests weakens or soil carbon release increases, forests may shift from carbon sinks to carbon sources, which could further increase atmospheric CO_2_ concentrations [4]. Therefore, plant activity is a key process linking carbon uptake and release; it is also an important factor influencing variability and uncertainty in estimates of ecosystem carbon cycling [5]. Accordingly, evaluating the response patterns of carbon uptake and release to changes in plant activity, together with the responses of microbial processes closely associated with soil carbon release, is important for improving our understanding of forest carbon cycling and enhancing predictive capacity under climate change [6].

Forests are a major carbon sink, and approximately half of forest carbon is stored in soil [7]. However, a substantial portion of the carbon stored in forest soils is released to the atmosphere as soil respiration (Rs) [8]. Thus, forests can function both as carbon sinks that absorb carbon and as carbon sources that release carbon through Rs. Forest plants absorb carbon through photosynthesis and supply assimilated carbon to soils in the form of litterfall, root biomass, and root exudates, thereby mediating organic matter accumulation and decomposition processes [9]. These processes can vary with plant activity levels such as photosynthesis, growth, and transpiration, which influence carbon inputs to soil and the resulting intensity of decomposition and respiration [10]. However, uncertainties remain regarding the mechanisms by which plant activity regulates Rs and the factors that determine the responsiveness of Rs [11]. Uncertainties also remain regarding the turnover time of atmospheric carbon as it passes through plants and soils before being released back to the atmosphere [12]. Reducing these uncertainties is a key research challenge for quantitatively projecting variability in ecosystem carbon cycling.

Rs can be partitioned into autotrophic respiration (Ra) derived from roots and the rhizosphere and heterotrophic respiration (Rh) derived from microbial decomposition [13,14]. However, because Rs is measured as an integrated flux of Ra and Rh, it is possible that the relative sensitivity and contribution of Ra and Rh may differ in response to changes in plant activity [15]. In particular, Ra is directly linked to plant activity, because plants transfer a portion of the carbon assimilated through photosynthesis belowground, and this carbon contributes to Ra through root respiration [16]. In contrast, increased plant activity can enhance the input of organic substrates from roots to soil, such as root exudates, which can increase substrate availability for soil microorganisms, as reflected in variability in Rh [17]. Accordingly, although recent soil respiration studies have considered differences in the responsiveness of Ra and Rh, substantial uncertainty remains regarding the time lag between changes in plant activity and their manifestation in Ra and Rh [18]. Therefore, to evaluate the direct and indirect influences of changes in plant activity on soil respiration components, it is necessary to partition Rs into Ra and Rh and to consider the response lags of both components [19].

In addition, increases in photosynthesis can intensify transpiration and increase plant water demand [20]. Photosynthesis reflects carbon assimilation and can be directly linked to the Ra pathway through the belowground transfer of assimilated carbon [21]. In contrast, sap flux density reflects the intensity of water transport and transpiration and represents changes in plant water balance; it may be indirectly associated with Rh or the environmental response of Rs by mediating changes in soil moisture conditions and microbial processes [22]. Therefore, sap flux density can be used as an indicator of plant activity distinct from photosynthesis, a carbon assimilation indicator, and it is useful for representing the hydrological aspect of plant activity [23].

Temperate deciduous broadleaf forests often show pronounced seasonal variation in plant activity [24]. This ecological characteristic provides a useful context for examining how plant activity is associated with changes and lagged responses in soil respiration components. Accordingly, this study aimed to use sap flux density and estimated photosynthesis as indicators of plant activity in a temperate deciduous broadleaf forest to compare the response patterns and time lags of soil respiration components (Rs, Rh, and Ra). Considering that Rs is observed as an integrated flux and that plant activity signals may be diluted, we partitioned Rs into Rh and Ra and aimed to compare how their association patterns and response lags differed in relation to plant activity. Furthermore, using high-temporal-resolution data, we conducted lagged correlation analyses to quantify the time lags between changes in estimated photosynthesis and sap flux density and the responses of Rs, Rh, and Ra. In particular, we focused on whether variability in soil respiration was linked to plant activity represented by estimated photosynthesis and sap flux density, as well as whether this linkage differed between Rh and Ra. We hypothesized that soil respiration would be associated with plant activity and that this association and its temporal lag would be more evident in autotrophic respiration than in heterotrophic respiration.

## 2. Results

### 2.1. Variation in Environmental Conditions and Canopy Structure

**Figure 1:**
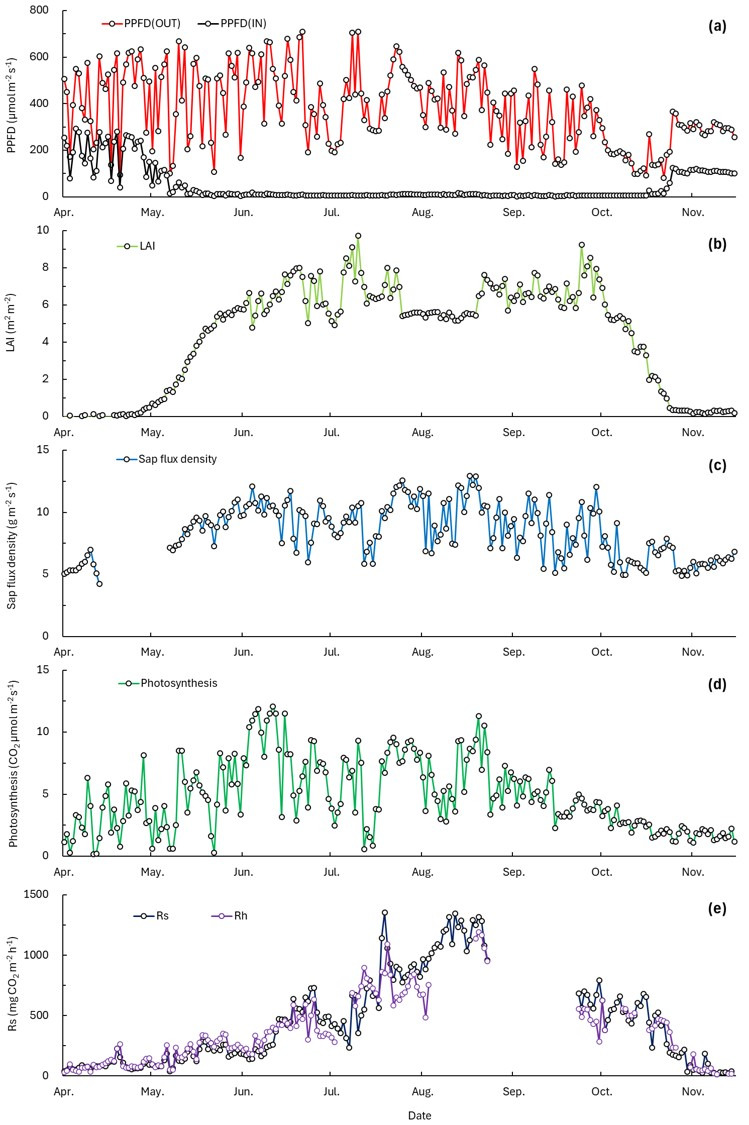
Seasonal dynamics of light environment, canopy structure, plant activity, and soil respiration from April to November. (**a**) PPFD above the canopy (OUT) and within the canopy (IN). (**b**) LAI. (**c**) Daily sap flux density. (**d**) Daily photosynthesis. (**e**) Rs and Rh. Rs data were unavailable from 26 August to 24 September because of instrument malfunction. Rh data were unavailable from 6 August to 20 August and from 26 August to 24 September.

Photosynthetic photon flux density (PPFD) was measured within the forest (IN) and at 21 m height on the ecological tower (OUT). During the study period from April to November, PPFD outside the forest averaged 379.0 ± 159.9 µmol m^−2^ s^−1^, whereas PPFD inside the forest averaged 46.1 ± 72.6 µmol m^−2^ s^−1^, representing an 87.8% reduction relative to outside conditions (Figure 1a). Air temperature (Ta) averaged 15.7 °C outside the forest and 14.9 °C inside the forest, with a mean difference of 0.8 °C. However, this difference began to increase from April and reached more than 3.5 °C during July, when air temperature was highest.

To quantify changes in canopy structure associated with plant growth and to evaluate variations in photosynthesis and plant activity by estimating PPFD within the canopy, plant area index (PAI) was measured. PAI was measured manually, and the relationship between canopy gap fraction and the light extinction coefficient (K) was derived using PPFD data (Figure S1). The reconstructed LAI showed good agreement between observed and estimated values (*R*^2^ = 0.887, RMSE = 0.925, MAE = 0.734, *n* = 6), and the derived relationship was used to estimate K, which showed an average value of 0.94 ± 0.28. PAI remained below 1 before June and increased thereafter. After May, when leaf development increased, peak PAI heights were distributed within the 5.15–7.85 m range aboveground, with most peaks occurring around 6 m. From June to September, 65.0% to 86.8% of total PAI was concentrated above 18 m height (Figure S2), indicating that canopy development became increasingly concentrated in the upper canopy during the main growing season.

Leaf area index (LAI) was calculated by subtracting wood area index (WAI) from PAI. LAI began to show positive values around April 20 and increased from late April. LAI then declined in October and remained below 1 by November (Figure 1b).

During the study period, sap flux density averaged 8.4 ± 2.2 g m^−2^ s^−1^, ranging from a minimum of 4.2 g m^−2^ s^−1^ to a maximum of 13.0 g m^−2^ s^−1^ (Figure 1c). Over the same period, photosynthesis averaged 4.8 ± 2.9 µmol CO_2_ m^−2^ s^−1^, increasing from April and declining after September (Figure 1d). Photosynthesis also showed substantial variability, with larger fluctuations observed on rainy or cloudy days. The photosynthesis estimation model showed agreement between observed and estimated values (*R*^2^ = 0.896, RMSE = 1.200, MAE = 0.888, n = 118, Figure S3).

Soil respiration (Rs) averaged 439.4 ± 368.0 mg CO_2_ m^−2^ h^−1^, whereas heterotrophic respiration (Rh) averaged 361.1 ± 272.9 mg CO_2_ m^−2^ h^−1^. Both Rs and Rh increased from April and declined after September, exhibiting similar seasonal patterns (Figure 1e).

### 2.2. Relationship Between Sap Flux Density and Environmental Factors

**Figure 2:**
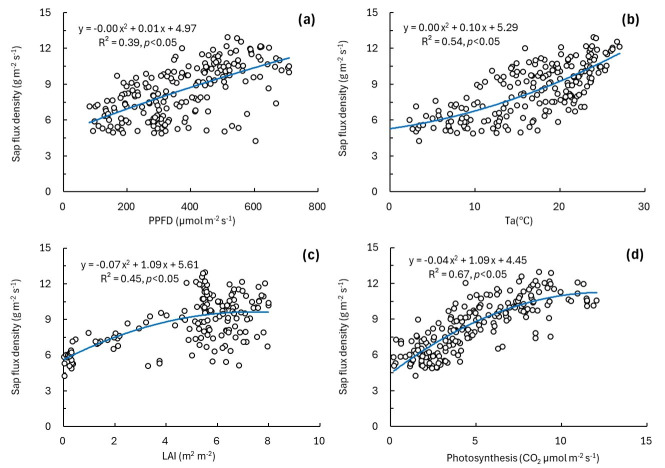
Relationships between sap flux density and environmental factors. (**a**) PPFD, (**b**) Ta, (**c**) LAI, and (**d**) photosynthesis.

**Figure 3:**
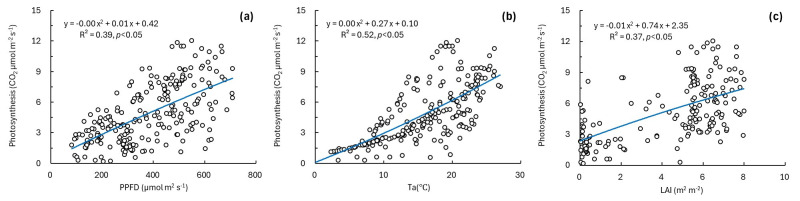
Relationships between photosynthesis and environmental factors. (**a**) PPFD, (**b**) Ta, and (**c**) LAI.

We used sap flux density and estimated photosynthesis as indicators of plant activity and evaluated the relationships between each indicator and environmental factors (PPFD, Ta, and LAI) using regression analyses (Figure 2 and Figure 3). Sap flux density showed nonlinear increasing relationships with PPFD, Ta, and LAI (Figure 2a–c), with coefficients of determination (*R*^2^) of 0.39 for PPFD, 0.54 for Ta, and 0.45 for LAI. In addition, sap flux density was positively related to photosynthesis (Figure 2d, *p* < 0.05), with the highest *R*^2^ among the tested variables (*R*^2^ = 0.67). Photosynthesis also increased with increasing PPFD, Ta, and LAI (Figure 3), with *R*^2^ values of 0.39 for PPFD, 0.52 for Ta, and 0.37 for LAI. All relationships between the plant activity indicators and environmental factors were significant (*p* < 0.05). Overall, the two plant activity indicators exhibited similar response patterns to variations in PPFD, Ta, and LAI.

### 2.3. Relationships Between Plant Activity Indicators and Rs, Rh, and Ra

**Figure 4:**
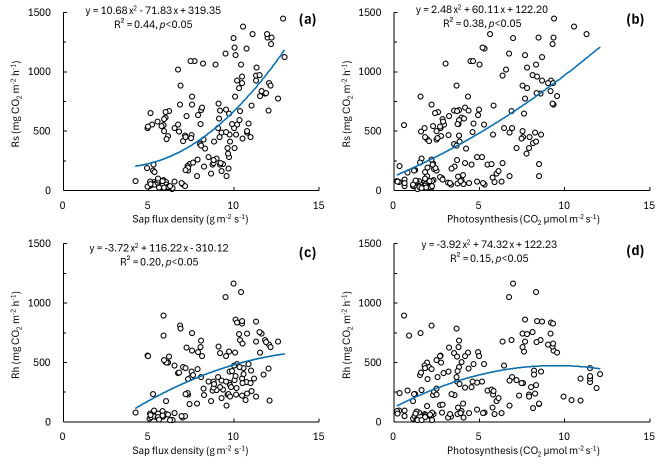
Relationships of soil respiration components with plant activity indicators. (**a**) Relationship between Rs and sap flux density, (**b**) relationship between Rs and photosynthesis, (**c**) relationship between Rh and sap flux density, and (**d**) relationship between Rh and photosynthesis.

The responses of Rs and Rh were analyzed using sap flux density and photosynthesis as indicators of plant activity (Figure 4). Rs increased with sap flux density (adjusted *R*^2^ = 0.42, RMSE = 283.77, Figure 4a) and photosynthesis (adjusted *R*^2^ = 0.37, RMSE = 294.19, Figure 4b), and both relationships were significant (*p* < 0.001). In contrast, Rh showed weaker relationships with sap flux density and photosynthesis, with adjusted *R*^2^ values of 0.19 and 0.13 and RMSE values of 232.02 and 241.24, respectively, although both relationships remained significant (*p* < 0.001; Figure 4c,d). The slope estimates and their 95% confidence intervals are summarized in Table S1. Overall, Rs showed stronger relationships with the plant activity indicators than Rh.

Ra estimated from Rs and Rh increased with sap flux density and photosynthesis (adjusted *R*^2^ = 0.36 and 0.29, RMSE = 78.42 and 77.47, respectively; Figure S4), and both relationships were significant (*p* < 0.001). The corresponding 95% confidence intervals are also provided in Table S1. However, because Ra was obtained via subtraction, these relationships should be interpreted with caution.

### 2.4. Time Lag Responses of Rs, Rh, and Ra to Changes in Plant Activity

**Figure 5:**
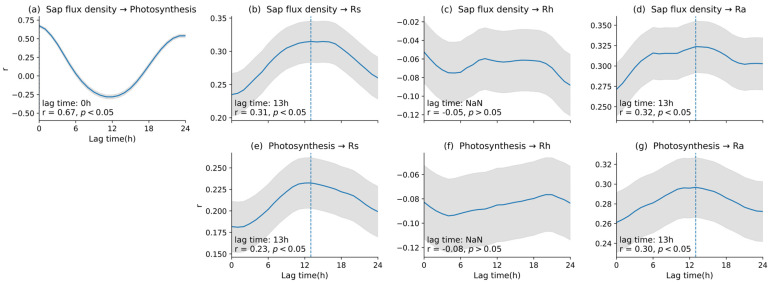
Time lag relationships between plant activity indicators and soil respiration components. Cross-correlation coefficients (r) are shown as a function of lag time for (**a**) sap flux density and photo-synthesis, (**b**) sap flux density and Rs, (**c**) sap flux density and Rh, (**d**) sap flux density and Ra, (**e**) photosynthesis and Rs, (**f**) photosynthesis and Rh, and (**g**) photosynthesis and Ra. Dashed lines indicate the lag time at which the maximum correlation was observed. Shaded areas indicate confidence intervals.

To examine response lags in soil respiration components associated with plant activity, we conducted a lagged cross-correlation analysis using high-temporal-resolution data for sap flux density and estimated photosynthesis, and for Rs, Rh, and Ra (Figure 5). Sap flux density and photosynthesis showed the highest correlation at a lag of 0 h (*r* = 0.67, *p* < 0.05), indicating that changes in the two variables occurred nearly simultaneously (Figure 5a). The relationship between sap flux density and Rs exhibited a maximum correlation at a lag of 13 h (*r* = 0.31, *p* < 0.05; Figure 5b), and sap flux density and Ra also showed a significant positive correlation at a lag of 13 h (*r* = 0.32, *p* < 0.05; Figure 5d). In contrast, Rh showed no significant lag with sap flux density, and the correlation was not significant (*r* = −0.05, *p* > 0.05; Figure 5c). However, although daily-scale regression analyses indicated significant relationships between Rh and both sap flux density and photosynthesis (adjusted *R*^2^ = 0.19 and 0.13; *p* < 0.001), no significant correlations were detected between Rh and the plant activity indicators in the lag-aware, high-temporal-resolution analysis. This discrepancy suggests that the daily-scale relationships may reflect shared covariation with environmental conditions or broader temporal patterns, rather than a distinct short-term lagged response of Rh to plant activity. For photosynthesis, Rs and Ra also showed significant positive correlations at a lag of 13 h (Rs: *r* = 0.23, *p* < 0.05; Ra: *r* = 0.30, *p* < 0.05; Figure 5e–g), whereas Rh showed no significant relationship (*r* = −0.08, *p* > 0.05; Figure 5f). The detected 13 h lags for Rs and Ra remained significant when significance was evaluated using the 24 h block-permutation test (Table S2), supporting the robustness of lag detection under temporally structured data.

## 3. Discussion

### 3.1. Characteristics of Plant Activity Variability

CO_2_ is widely recognized as an essential carbon source required for plants to synthesize organic compounds through photosynthesis [24]. Plants absorb atmospheric CO_2_ and convert it into biomass, and they contribute to ecosystem carbon fluxes by supplying carbon to soils through the belowground transport of assimilates and through root and rhizosphere respiration [10]. Changes in plant activity also alter plant carbon allocation, thereby regulating the timing and magnitude of carbon transfer from the atmosphere to the soil [25]. Quantifying these carbon fluxes is essential for predicting changes in plant physiology and ecosystem carbon cycling under global warming and for improving the accuracy of such predictions.

Accordingly, in this study, we characterized plant activity variability using indicators such as LAI, sap flux density, and photosynthesis. LAI was measured across canopy layers, and photosynthesis was measured in the upper canopy (18 m) to represent CO_2_ uptake characteristics of upper canopy leaves, considering the vertical distribution of LAI (Figure S2). LAI increased from April, reached a maximum during summer, and then declined from late October, and the difference in PPFD between outside and inside the forest tended to increase as LAI increased (Figure 1a,b). This pattern indicates a shading effect associated with leaf growth and canopy development and suggests the seasonal enhancement of plant activity during the growing season [26]. LAI is known to influence photosynthesis by modifying light absorption and light use efficiency [27,28], and, in this study, photosynthesis increased with increasing PPFD. In addition, photosynthetic responses to changes in Ta were observed (Figure 3), consistent with the influence of Ta on photosynthetic rates via enzymatic kinetics and stomatal regulation [29].

Sap flux density reflects plant transpiration and stomatal activity [30]. In this study, sap flux density showed positive relationships with PPFD, Ta, LAI, and photosynthesis, and its temporal pattern was generally consistent with photosynthetic responses to environmental factors (Figure 2 and Figure 3). These patterns are consistent with previous studies showing that sap flux density reflects plant water use status, as regulated by environmental conditions and stomatal activity [31,32,33,34]. Therefore, sap flux density was interpreted as an indicator of plant activity related to plant water use status, whereas estimated photosynthesis was used to reflect carbon assimilation.

### 3.2. Relationship Between Plant Activity and Soil Respiration

Rs showed positive relationships with sap flux density and photosynthesis, and it tended to covary with fluctuations in the plant activity indicators (Figure 4a,b). This pattern suggests that Rs variability was associated with plant activity, potentially in connection with assimilate supply and belowground carbon allocation. However, because Rs comprises Rh and Ra, the magnitude of the responses and the response lags of Rh and Ra to changes in plant activity may differ. Therefore, to determine whether the association with plant activity was primarily attributable to Ra or also reflected in Rh, we partitioned Rs into Rh and Ra and discussed their responses to plant activity separately.

#### 3.2.1. Relationship Between Plant Activity and Root Respiration

Ra, calculated as the difference between Rs and Rh, showed positive relationships with sap flux density and photosynthesis, with coefficients of determination (*R*^2^) of 0.37 and 0.30, respectively (Figure S4). Ra was significantly related to both sap flux density and photosynthesis (*p* < 0.05). These patterns are consistent with a potential link between plant activity and Ra, although the relationships should be interpreted cautiously. Photosynthetically derived assimilates may be transported belowground and may influence root growth and root exudation [35], which could in turn be associated with root and rhizosphere respiration [36]. However, because Ra was derived from the difference between the estimated values of Rs and Rh, occasional negative Ra values indicate uncertainty in the partitioning procedure. In addition, although subtraction-based partitioning has been widely used [37,38], this approach assumes that soil respiration measured in trenched plots represents Rh and that microbial conditions remain comparable between trenched and untrenched soils [39]. In practice, root exclusion can alter soil moisture, soil temperature, substrate supply, microbial community composition, microbial biomass, and microbial activity, which may influence Rh estimates and propagate uncertainty into Ra [40,41]. Therefore, the observed Ra patterns should be regarded as suggestive rather than conclusive evidence of autotrophic respiration responses to plant activity and their contribution to soil respiration variability.

#### 3.2.2. Relationship Between Plant Activity and Heterotrophic Respiration

Rh showed significant but relatively weak relationships with sap flux density and photosynthesis in the daily scale regression analysis, whereas no significant lagged relationship was detected in the high-temporal-resolution cross-correlation analysis (Figure 4c,d). This contrast between analyses suggests that the significant daily-scale relationships may not reflect a distinct short-term response of Rh to plant activity alone. Instead, they may reflect indirect coupling through shared environmental controls or broader seasonal and diel covariation [42]. At the daily scale, significant associations may reflect shared temporal variation with plant activity and other concurrent influences, rather than a distinct short term lagged response [43]. Taken together, these results suggest that the significant relationships observed in the daily scale regression analysis more likely reflect broader temporal covariation or indirect coupling than a clear immediate response of Rh to plant activity. In addition, because Rh was measured in a trenched plot, its estimates may also have been affected by trenching induced changes in soil moisture, soil temperature, and microbial conditions. Uncertainty in the estimated photosynthesis may have affected the strength of its reported relationships with Rs, Rh, and Ra, and this limitation should be considered when interpreting the results.

### 3.3. Response Characteristics of Soil Respiration Components to Plant Activity

To quantify the time lag between changes in plant activity (sap flux density and photosynthesis) and the responses of soil respiration components (Rs, Rh, and Ra), we conducted a lag correlation analysis (Figure 5). Because data aggregated at daily resolution can average out short term variability and weaken lag signals, we analyzed lag responses using high temporal resolution data.

Sap flux density and photosynthesis changed almost simultaneously within the same diel cycle, whereas Rs showed a maximum correlation at a lag of approximately 13 h relative to plant activity (Figure 5). Previous studies have reported contrasting lag responses between plant activity and soil respiration. Almost no detectable lag was observed at the daily scale in a drought-affected Mediterranean forest, whereas a lag of 0–4 h was reported in a coastal wetland and 7–12 h in an oak woodland ecosystem [9,44,45]. Compared with these studies, the lag observed in our temperate deciduous broadleaf forest was approximately 13 h. Such differences in lag duration may reflect site-specific environmental conditions, including tree height, temperature, and moisture [46]. Nevertheless, the response observed at our site fell within the broader range reported for forest ecosystems. These findings suggest that total soil respiration covaried with plant activity represented by both photosynthesis and sap flux density, and that the detected lag pattern was more consistent with the partitioned Ra estimate than with Rh. Ra also showed a significant correlation at the same 13 h lag as Rs. This pattern is consistent with the possibility that recent assimilate supply contributed to the lagged response, although alternative explanations should also be considered, including diel covariation with temperature, water-mediated processes such as hydraulic redistribution, and residual temporal autocorrelation.

In contrast, Rh showed no clear lag correlation with plant activity. This suggests that, over short time scales, Rh may be influenced more by concurrent local conditions than by changes in plant activity [47]. Accordingly, the significant relationships observed in the daily scale regression analysis likely reflect broader temporal covariation or indirect coupling, rather than a detectable short-term lagged response of Rh to plant activity. Overall, the lagged response of Rs to plant activity was more consistent with the partitioned Ra estimate than with Rh, and the approximately 13 h lag is consistent with the possibility that recent assimilates influenced belowground respiration with a temporal delay. Because this study was conducted at a single temperate deciduous forest site during one growing season, these relationships and lag patterns may reflect site- and year-specific conditions.

## 4. Materials and Methods

### 4.1. Site Description

**Figure 6:**
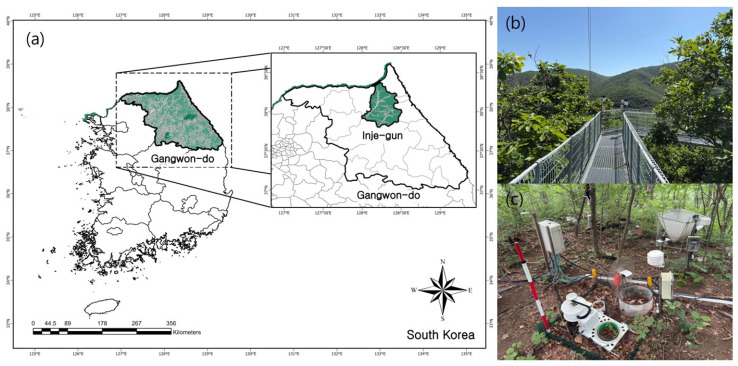
Location of the study site on Mt. Jeombong, South Korea (128°25′–128°30′ E, 38°0′–38°5′ N), and photographs of the site. (**a**) Map of the study site, with the green polygon indicating the forested area. (**b**) View from the ecological tower. (**c**) View within the forest stand.

The study was conducted on Mt. Jeombong (128°25′–128°30′ E, 38°0′–38°5′ N), situated in the middle of the Korean Peninsula (Figure 6). Climatically, it falls within the temperate deciduous broadleaf forest zone. The average annual air temperature of Mt. Jeombong is 9.9 °C [48]. A previous study reported that the precipitation in the research site from April to November was 1426.2 ± 412.3 mm [49]. The dominant species of the research site are *Quercus mongolica* in the canopy and *Acer pseudosieboldianum* in the understory. Mt. Jeombong is an area suitable for long-term ecological research due to its well-preserved ecosystem; thus, multiple ecological studies have been conducted in this area [50,51].

The research site is located on Mt. Jeombong, and the altitude of the research site is 786 m. The slope gradient is 5° and the meridian angle is 32°. The wind direction at the research site was mainly southwest. In this study, the biomass carbon stock of the research site was estimated to be 325.8 t C ha^−1^, and carbon accumulation from the organic horizon to a depth of 30 cm was measured at 3.84 ± 0.71 t C ha^−1^.

### 4.2. Soil Respiration

In this study, we measured Rs using the automatic open–closed chamber (AOCC) system, which is one of the automatic Rs measurement methods.

The AOCC system measured Rs using a closed dynamic method. The AOCC system consists of the chamber, pump, and timer unit. The chamber had a height of 20 cm and a diameter of 40 cm. Rs measurements were conducted when the signal from the timer unit triggered chamber closure, and, at the same time, the pump sent air to each chamber and measured the CO_2_ concentration in the air returning from the chamber through an infrared gas analyzer (IRGA). Six chambers were installed on the subplot of the ridge, which is 12 m square in size. One chamber was established on the trench plot, two were on the west ridge, and the remaining chambers were established on the east ridge. Before the measurements began, the trench plot was stabilized for over one year after its establishment and was used in a previous study with continuous aboveground vegetation removal. The trench was excavated to a depth of 1 m, and the plot was inspected monthly. These procedures were intended to minimize short term disturbance after trench installation and to maintain root exclusion during the measurement period. For analyses of the relationship between soil respiration and plant activity, Rh was obtained from the single AOCC chamber installed in the trench plot, whereas Rs was represented by the mean of three AOCC chambers installed in the east ridge, where the sap flow equipment was also located. Ra was calculated as Rs minus Rh. The trench plot and the east ridge chambers were located within 3 m of each other. The trench plot was situated on a 4° slope, whereas the east ridge chambers were located on a 6° slope. During the study period, the mean soil temperature was 14.5 ± 5.4 °C in the trench plot and 14.6 ± 5.4 °C in the east ridge chambers, and this difference was not significant (*p* > 0.05). Mean soil moisture was 22.7 ± 3.5% in the trench plot and 24.7 ± 4.7% in the east ridge chambers. Litter accumulation was 617.0 g m^−2^ in the trench plot and 733.7 g m^−2^ in the east ridge chambers. The prevailing wind direction was from the trench plot toward the east ridge chambers. These observations indicate that the two locations were close and broadly comparable in terms of field conditions, although they were not identical microsites. However, we did not directly correct for trench-induced changes in soil moisture, soil temperature, or microbial dynamics, and we did not independently quantify additional soil physicochemical differences between the two microsites. These limitations were therefore considered when interpreting Rh, Ra, and the partitioned fluxes. Each chamber was alternately closed at 30 min intervals for 5 min to measure Rs. The CO_2_ concentration was measured using a Li-820 (Licor Inc., Lincoln, NE, USA), a type of IRGA, and the data were collected using a CR1000 data logger once a minute (Campbell Scientific Inc., Logan, UT, USA). Rs measurements using the AOCC system were conducted from April 2025 to November 2025. Rs was calculated using (Equation (1)):

(1) Rs (mg CO_2_ m^−2^ h^−1^) = (*a* × *V* × *ρ*) × *A*^−1^

In this formula, a refers to the increasing rate of the CO_2_ concentration per unit time (ppm min^−1^), V indicates the volume of the collar (m^3^), *ρ* denotes the density of CO_2_ (mg m^−3^), and A is the surface area of soil enclosed within the automatic Rs chamber (m^2^). Invalid observations and data gaps due to malfunctions in IRGA and power supply were excluded from the analysis. The proportion of excluded observations was 15.8% in Rs and 33.3% in Rh:

(2) Ra (mg CO_2_ m^−2^ h^−1^) = Rs − Rh

Ra was calculated as the difference between Rs and Rh using Equation (2). Because Ra was derived from two independently measured fluxes, uncertainty in both Rs and Rh could propagate into the Ra estimate. Negative Ra values occurred in 22.2% of the hourly observations (n = 918 of 4133) and were interpreted as reflecting partitioning uncertainty rather than biologically meaningful negative autotrophic respiration. These values were retained to avoid bias associated with truncation, and their frequency was evaluated at the hourly scale, together with the uncertainty analysis described below:

(3) $$u\left(Ra\right)\;=\;\sqrt{{\left[u\left(Rs\right)\right]}^{2}+{\left[u\left(Rh\right)\right]}^{2}}$$

The uncertainty of Ra was calculated using Equation (3), where $u\left(Ra\right)$, $u\left(Rs\right)$, and $u\left(Rh\right)$ denote the uncertainties associated with Ra, Rs, and Rh, respectively. Because Rh was measured using a single chamber, $u\left(Rh\right)$ could not be quantified directly. Therefore, the present uncertainty analysis represents a conservative estimate based only on *u*(*Rs*). Under this assumption, 32.29% of negative Ra values overlapped with zero within the 95% uncertainty interval. This result indicates that some of the negative Ra values may fall within the range of measurement uncertainty. However, if $u\left(Rh\right)$ had also been quantified and included, the proportion of negative $u\left(Ra\right)$ values overlapping zero could have remained unchanged or increased.

### 4.3. Environmental Factors

Rainfall, PPFD, and Ta were measured using data loggers. PPFD was measured using a light sensor (M-NDVI, Onset, Bourne, MA, USA). PPFD was measured above the canopy and within the forest to characterize seasonal changes in the light environment associated with canopy development. To ensure continuous data collection, PPFD was additionally measured using a CS310 sensor (Campbell Scientific Inc., Logan, UT, USA). Measurements of PPFD were conducted at the ridge, the trench plot of the ridge, and at the 21 m level of the ecological tower. Ta was measured using a temperature sensor (S-TMB-M002, Onset, Bourne, MA, USA), and Ta data were recorded using a HOBO Micro Station (Onset, Bourne, MA, USA). PPFD measured using the M-NDVI sensor was recorded using a HOBO Micro Station (Onset, Bourne, MA, USA), whereas PPFD measured using the CS310 sensor was recorded by a CR800 data logger (Campbell Scientific Inc., Logan, UT, USA).

### 4.4. Leaf Area Index

LAI is defined as the sum of the one-sided leaf area of the plant canopy per unit surface area [52]. We measured the canopy gap fraction using an optical method with the LAI-2000 Plant Canopy Analyzer. In this study, the optical estimate was treated as the plant area index, which includes both leaves and woody elements. The wood area index was estimated from observations conducted before leaf out (April 2025) and was assumed to be constant during the study period. LAI was calculated as LAI = PAI − WAI. We conducted measurements once a month from April 2025 to November 2025 at the research site. To analyze the vertical structure of the forest canopy along with the total forest canopy, measurements were conducted at heights of 18 m, 15 m, 12 m, 6 m, and ground. Measurements were conducted three times at each height. Because vertical measurements were not available in October 2025, the relationship used for continuous LAI estimation was derived from the measurements obtained from April to September. This relationship was then applied to the above- and below-canopy PPFD data to reconstruct the continuous LAI time series, including October.

Manual LAI measurements have limitations in capturing phenological changes in vegetation due to their low frequency. Thus, we substituted the monthly LAI data (calculated as LAI = PAI − WAI) and above- and below-canopy PPFD data into the LAI formula. Using this formula, LAI was continuously estimated. LAI was estimated using Equation (4) [53]:

(4) I = I_0_ × e^−KLAI^

In this formula, I refers to the PPFD below the canopy, I_0_ indicates the PPFD above the canopy, and K denotes the light extinction coefficient. I and I_0_ use the PPFD data recorded in the HOBO Micro Station (Onset, Bourne, MA, USA). Since a previous study reported that light extinction coefficients in *Q. mongolica* forests differ notably across plant phenology [54], the relationship between canopy light transmission and the light extinction coefficient was estimated using environmental data at the time of LAI measurement [55].

### 4.5. Photosynthesis

Photosynthesis was quantified by measuring the photosynthetic rate under controlled light and temperature conditions and then obtaining an equation. For photosynthesis measurements, we used the Li-6400XT (LI-COR Inc., Lincoln, NE, USA), which is widely used in photosynthetic research. The Li-6400XT consists of a chamber unit and a main console. The photosynthesis measurement was performed after placing the leaf in the chamber and stabilizing it at a PPFD of 1500 μmol m^−2^ s^−1^ at a fixed leaf temperature. This light condition is also the standard used in previous studies conducted in *Q. mongolica* forests [54]. Stabilization was performed at a certain leaf temperature over 10 min, and then measurements were performed by sequentially lowering the PPFD to 1400, 1200, 1000, 800, 600, 400, 200, 100, 50, and 0 μmol m^−2^ s^−1^ in turn. Photosynthesis was measured using the chamber IRGA by comparing the CO_2_ concentrations between the sample and reference air streams. During this process, fluctuations in humidity, a crucial factor affecting photosynthesis, could inhibit photosynthesis. Hence, humidity was maintained at 50–60% [56]. After the measurement was completed at one leaf temperature, we changed the leaf temperature, and the measurement was carried out by stabilizing for 10 min at PPFD 1500 μmol m^−2^ s^−1^. Photosynthesis measurements were conducted once per month during the leaf-bearing period from May to October 2025, yielding six measurement campaigns in total. Measurements were performed on *Quercus mongolica* leaves collected from the upper canopy at 18 m on the ecological tower, and three replicate leaves were measured for each campaign. Only undamaged leaves longer than 10 cm were selected, and young developing leaves or senescing leaves were avoided. In addition, only dry and uncontaminated leaves were used for the measurements. Photosynthesis measurements were conducted across at least three different leaf temperature levels during each campaign.

Photosynthesis was calculated using the non-rectangular hyperbola (NRH) formula of Ye et al. [57]. The NRH model was fitted using Equation (5):

(5) Net photosynthetic rate = α_p_ × ((1 − β_p_ × I)/(1 + γ_p_ × I)) × I − R_light_

In this formula, α_p_ refers to the initial slope of the photosynthesis–light response curve, β_p_ indicates the photoinhibition coefficient, γ_p_ denotes the photo saturation coefficient, I is the photosynthetic photon flux density, and R_light_ is the non-photorespiratory mitochondrial CO_2_ release in light. As NRH models explicitly represent light conditions, they have been parameterized according to phenological or physiological attributes, including leaf age [58]. Here, we estimated the photosynthetic rate by modifying parameters of the NRH equations using the Arrhenius equation [59] and adjusting respiration through the Q_10_ model [60]. The parameters were adjusted using the Arrhenius equation (Equation (6)):

(6) Parameter = Parameter_25_ × exp(((T_k_ − 298) × ΔHa)/(R × T_k_ × 298))

In this equation, Parameter_25_ refers to the parameter at 25 °C, T_k_ indicates the leaf temperature (K), ΔHa denotes the energy of activation (kJ mol^−1^), and R is the universal gas constant (8.314 J K^−1^ mol^−1^).

Photosynthesis measurements were conducted under controlled light (PPFD) and temperature conditions to estimate photosynthetic coefficients, which were subsequently applied to field PPFD and air temperature data to estimate continuous photosynthesis over the study period. Plant phenology also affects the photosynthesis rate. Therefore, photosynthesis was continuously estimated by applying equations derived from temporal measurements to the environmental data of the corresponding period. In this study, the resulting photosynthesis values were used as a continuous indicator of plant activity rather than as an independently validated canopy scale flux.

### 4.6. Sap Flux Density

Sap flow refers to the movement of water through the plant’s xylem, which is an important indicator of a plant’s transpiration and water use [61,62]. In this study, we used the SFM1 (ICT International, Australia), which measures the sap flow using the heat ratio method, a type of heat-pulse velocity method [61]. SFM1 measures the sap flow based on the differences in heat loss due to the flow of sap. It consists of a heater sensor, two temperature sensors, and a main console unit. After the SFM1 was installed on the sample plant, the heat sensor released heat at certain intervals, and the temperature sensors, which were installed below and at the bottom of the heat sensor, measured the heat loss. Sap flux density is calculated using this measured heat loss. To conduct this process precisely, the heater sensor is located between the two temperature sensors, which were installed vertically and equilibrated with the heater at 5 mm intervals. Using this method, we analyzed the temporal fluctuations in plant activity.

Prior to estimating sap flow, we collected tree samples using an increment borer and measured the dried density of the wood, as well as the moisture content of the sapwood, which are used in the estimations of thermal diffusivity. Calculations of thermal diffusivity are as follows [63,64]:

(7) k = (K × 10^6^)/(p × c)

In this equation, k refers to thermal diffusivity (mm^2^ s^−1^), K denotes the thermal conductivity (W m^−1^ K^−1^), c indicates specific heat capacity (J kg^−1^ K^−1^), and p is the density of the fresh sapwood (kg m^−3^). The corresponding K was calculated using Equation (8):

(8) K = (K_w_ × P_s_ + (K_s_ − K_w_) × m_c_ × P_b_)/P_s_

In this equation, K refers to thermal conductivity (W m^−1^ K^−1^), K_w_ denotes thermal conductivity of the dry wood matrix, K_s_ indicates thermal conductivity of water (0.5918 W m^−1^ K^−1^), P_b_ means density of the dried sapwood (g cm^−3^), and m_c_ is the moisture content of the sapwood (g g^−1^). K_w_ was calculated using Equation (9):

(9) K_w_ = 0.04182 × (21 − 20 × (1 − Pb × (0.667 + m_c_)))

In this equation, K_w_ refers to thermal conductivity of the dry wood matrix (W m^−1^ K^−1^), P_b_ is the density of the dried sapwood (g cm^−3^), and m_c_ denotes moisture content of the sapwood (g g^−1^). The specific heat capacity was calculated using Equation (10):

(10) c = (W_d_ × C_dw_ + C_s_ × (W_f_ − W_d_))/W_f_

In this equation, c refers to specific heat capacity (J kg^−1^ K^−1^), W_d_ denotes the dried weight of the sapwood, W_f_ indicates the fresh weight of the sapwood, and C_dw_ denotes the heat capacity of the dry wood matrix, and Cs refers to the heat capacity of the sap (4186 J kg^−1^ K^−1^).

The raw heat pulse velocity was calculated using the thermal diffusivity obtained using the above-described calculation process, the distance between the heater sensor and the temperature sensor, and the measured temperature increase of the two temperature sensors [61]. The thermal diffusivity was converted from mm^2^ s^−1^ to cm^2^ s^−1^ before calculating Vh. The raw heat velocity was calculated using Equation (11):

(11) V_h_ (cm h^−1^) = (3600 × k/x) × ln(V_1_/V_2_)

In this equation, V_h_ refers to raw heat pulse velocity, x denotes the distance between the heat sensor and the temperature sensor (cm), k means the thermal diffusivity (cm^2^ s^−1^), V_1_ indicates the temperature increase at the downstream, and V_2_ is the temperature increase at the upstream. The heat velocity obtained from Equation (10) was corrected using the wound factor. Sap flux density was calculated using the equation described in [65]:

(12) SFD = (P_b_/P_s_) × ((m_c_ × C_s_ + C_dw_)/C_s_) × u′

In this equation, SFD refers to sap flux density (cm^3^ cm^−2^ h^−1^), P_b_ denotes the density of the dry wood matrix (g cm^−3^), P_s_ indicates the density of the sap (1.0 g cm^−3^), and m_c_ is the moisture content of the sapwood. C_s_ refers to the heat capacity of the sap (4.186 J g^−1^ K^−1^), C_dw_ denotes the heat capacity of the dry wood matrix (1.380 J g^−1^ K^−1^), and u′ is the corrected sap velocity (cm h^−1^). The sap flux density calculated using Equation (11) was converted into the unit of g m^−2^ s^−1^ to facilitate comparison with previous studies conducted at the research site. Some of the above-described calculations were performed using Sap Flow Tool software version 1.5.1 (ICT International, Armidale, Australia).

### 4.7. Data Analysis

Plant physiological indicators were also statistically analyzed. Photosynthetic rate equations were obtained using nonlinear least squares (NLS) functions of R (version 4.4.2). Sap velocity was calculated using the Sap Flow tool software (ICT International, Australia). For the simple regression models relating plant activity indicators to respiration components, sample size (n), slope estimates, *p*-values, 95% confidence intervals, adjusted *R*^2^, and RMSE were additionally calculated (Table S1). In addition, the performance of the LAI reconstruction and photosynthesis estimation models was evaluated using **R*^2^*, RMSE, and MAE by comparing observed and estimated values (Figure S3). The relationships between plant activity indicators and respiration components were evaluated using simple linear regression to provide comparable estimates of association strength across variables over the observed range. To quantify time lags between plant activity and soil respiration components, AOCC values were treated as outliers only when they exceeded the corresponding nearby manual chamber observation three times and were therefore judged to reflect measurement errors rather than ecological variation. We removed outliers through data quality control and then used the CCF function in R (version 4.4.2) to perform time-lagged cross-correlation analyses between each plant activity indicator (sap flux density and estimated photosynthesis) and each soil respiration component (Rs, Rh, and Ra). For each variable pair, the peak lag was defined as the lag time with the maximum cross correlation coefficient. The number of paired observations, Pearson *p*-values, and block permutation *p*-values for each analysis are reported (Table S2). No additional detrending of seasonal trends was applied before the CCF analysis. To account for temporal autocorrelation and recurring within-day covariation among plant activity, soil respiration, and environmental conditions, the significance of the observed maximum cross correlation was assessed using a block permutation test with a 24 h block size, because the time series showed a clear diel cycle. For each variable pair, significance was evaluated by comparing the observed maximum cross correlation with the distribution obtained from permuting the time series in 24 h blocks, using the resulting empirical *p*-values. This approach was used to reduce the influence of recurring within-day patterns and temporal autocorrelation on lag detection.

## 5. Conclusions

This study evaluated relationships between plant activity indicators (LAI, sap flux density, and estimated photosynthesis) and soil respiration components (Rs, Rh, and Ra). During the growing season, changes in light and temperature conditions were associated with changes in the plant activity indicators, and sap flux density and photosynthesis covaried. Rs showed significant positive relationships with sap flux density and estimated photosynthesis. When Rs was partitioned, Ra appeared to show clearer associations with the plant activity indicators than Rh. However, because Ra was derived from trenching-based partitioning and subtraction, this pattern should be interpreted cautiously. Rh also showed significant but weak relationships in the daily-scale regression analysis, but no significant lagged relationship with plant activity was detected in the high-temporal-resolution analysis. This contrast suggests that the Rh associations likely reflected indirect or scale-dependent covariation rather than a clear short-term response to plant activity. Lagged correlation analysis further showed that Rs and Ra responded to plant activity indicators with a time delay of approximately 13 h. This pattern is consistent with the possibility of delayed belowground use of recent assimilates, but alternative explanations such as diel covariation in temperature, water mediated processes, or residual temporal autocorrelation should also be considered. Overall, variability in soil respiration showed temporal covariation with plant activity represented by both estimated photosynthesis and sap flux density, and the detected lag pattern was more consistent with the partitioned Ra estimate than with Rh. These findings suggest that longer-term observations across multiple years and contrasting ecosystems are needed to evaluate the broader generality of these relationships.

## Data Availability

The raw data supporting the conclusions of this article will be made available by the authors on request. The data are not publicly available due to institutional restrictions on sharing long term monitoring data collected at a protected forest research site.

## Supplementary Materials

The following supporting information can be downloaded at: https://www.mdpi.com/article/10.3390/plants15081175/s1, Figure S1: Relationship between canopy gap fraction and the light extinction coefficient (K) derived from PPFD data and manual LAI measurements. Figure S2: Vertical distribution of plant area index (PAI) along canopy height from April to September. Symbols indicate observed values at each height and dashed lines represent month specific linear fits. Figure S3: Comparison between observed and estimated values of (a) leaf area index (LAI) and (b) photosynthesis. Solid blue lines represent linear regression fits, and dashed gray lines indicate the 1:1 relationship. Figure S4: Relationships between Ra and plant activity indicators. (a) Ra and sap flux density and (b) Ra and photosynthesis. Solid lines represent fitted regression models. Ra was calculated as Rs minus Rh. Table S1: Summary of simple linear regression results for the relationships between plant activity indicators and respiration components. The table presents the slope (β), *p*-value, 95% confidence interval, adjusted *R*^2^, and RMSE for the relationships of sap flux density and photosynthesis with Rs, Rh, and Ra. Table S2: Peak lag times and cross correlation results between plant activity indicators (sap flux density and photosynthesis) and soil respiration components (Rs, Rh, and Ra). Peak lag is the lag time with the maximum cross correlation, and r is the corresponding correlation coefficient. n is the number of paired observations used after excluding missing values and outliers. Significance was evaluated using a block permutation test, and Pearson *p* values are also reported. NA indicates no significant lagged correlation.

## Abbreviations

The following abbreviations are used in this manuscript:

Rs	Soil respiration
Ra	Autotrophic respiration
Rh	Heterotrophic respiration
PPFD	Photosynthetic Photon Flux Density
CO_2_	Carbon dioxide
AOCC	Automatic open-closed chamber
IRGA	Infrared gas analyzer
Ta	Air temperature
LAI	Leaf area index
NRH	Non-rectangular hyperbola
NLS	nonlinear least squares
PAI	Plant area index
WAI	Wood area index
K	Light extinction coefficient
