# Supplementary material for: Differential Responses and Temporal Lags of Heterotrophic and Autotrophic Respiration to Plant Activity in a Forest Ecosystem
Source: Plants (Basel). 2026 Apr 10;15(8):1175. doi: 10.3390/plants15081175 (PMC13120146; doi:10.3390/plants15081175)
Supplement: Supplementary file 1 [file plants-15-01175-s001.zip › plants-4218344-supplementary.pdf]

Supplementary Materials

Differential responses and temporal lags of heterotrophic and autotrophic respiration to plant activity in a forest ecosystem

Dongmin Seo [1], Minyoung Lee [1], Youngsang Lee [2] and Jeaseok Lee [1*]

[1] Department of Biological Science, Konkuk University, Seoul 05029, Republic of Korea; adrea5957@konkuk.ac.kr (D.S.); my991004@naver.com (M.L.)
[2] Ecosystem Change Research Team, National Institute of Ecology, Seocheon, Republic of Korea; lys890309@nie.re.kr (Y.S.)
* Correspondence: jaeseok@konkuk.ac.kr

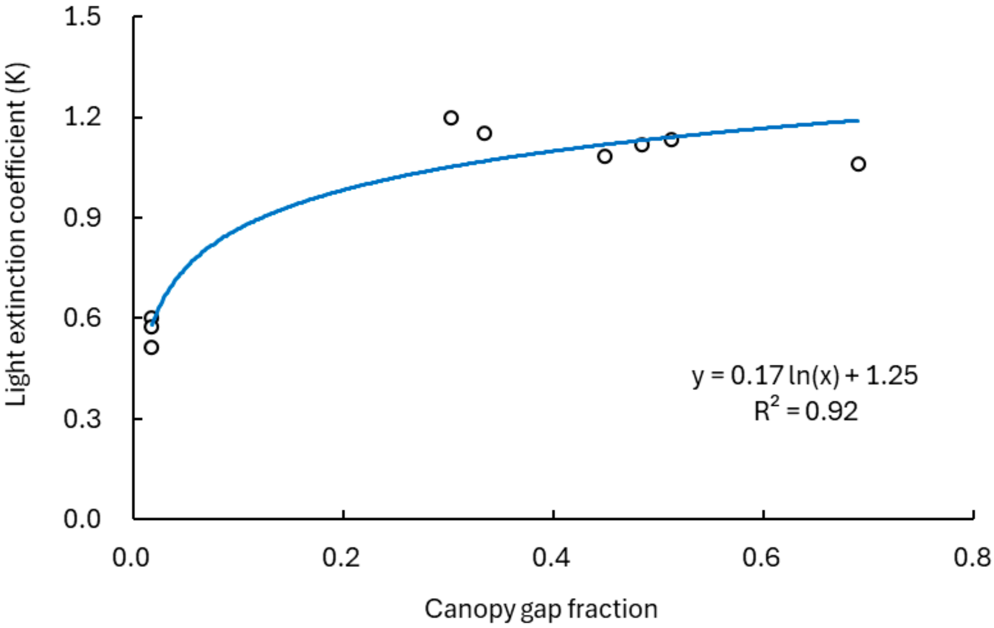

Figure S1. Relationship between canopy gap fraction and the light extinction coefficient (K) derived from PPFD data and manual LAI measurements.

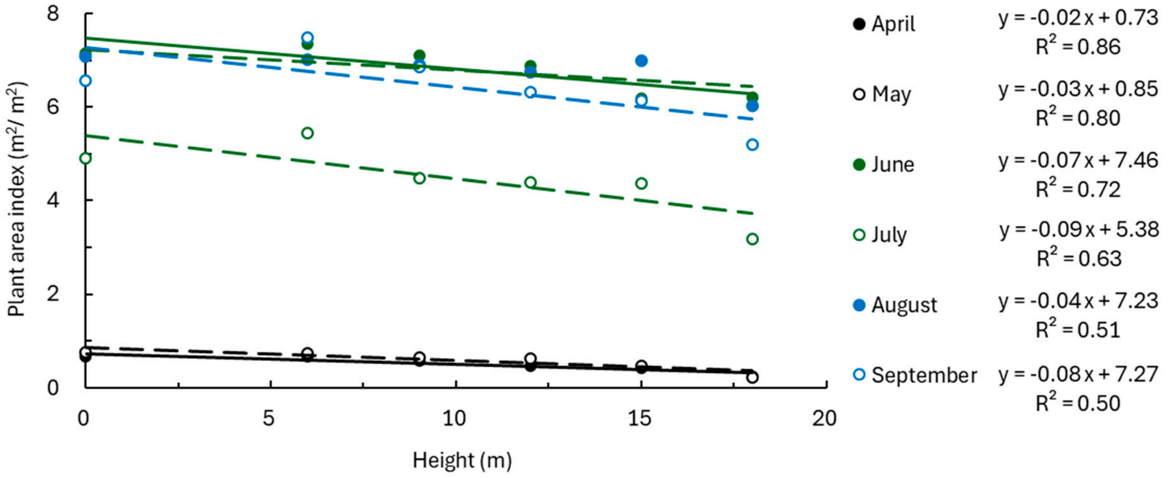

Figure S2. Vertical distribution of plant area index (PAI) along canopy height from April to September. Symbols indicate observed values at each height and dashed lines represent month specific linear fits.

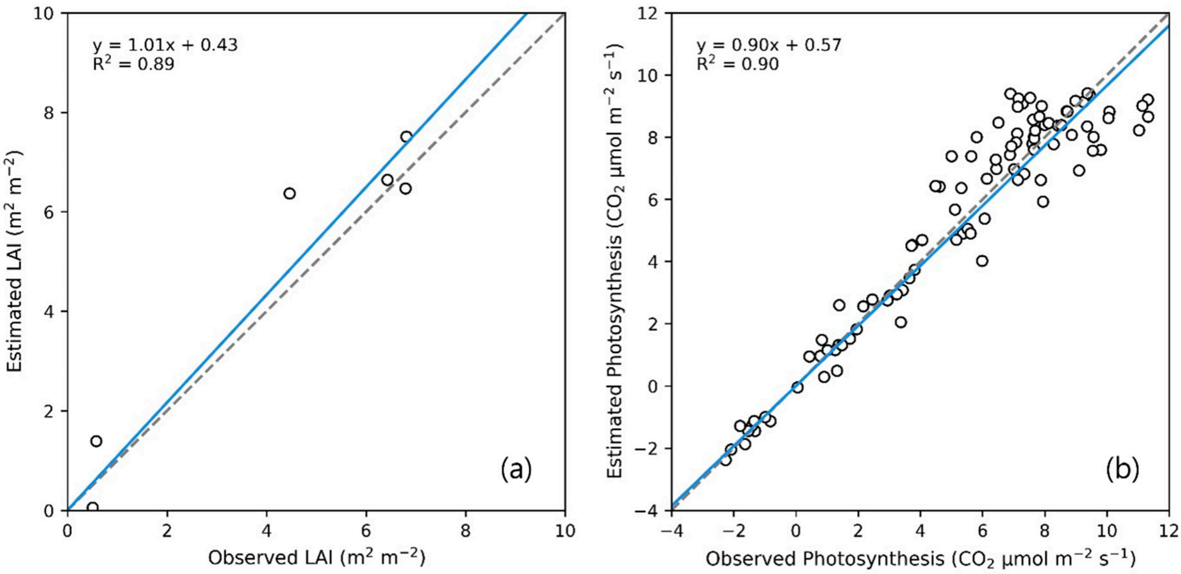

Figure S3. Comparison between observed and estimated values of (a) leaf area index (LAI) and (b) photosynthesis. Solid blue lines represent linear regression fits, and dashed gray lines indicate the 1:1 relationship.

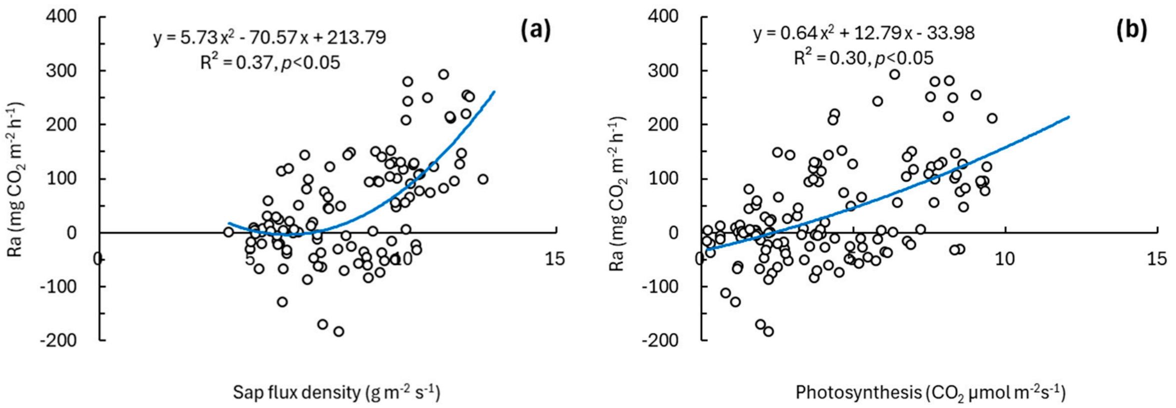

Figure S4. Relationships between Ra and plant activity indicators. (a) Ra and sap flux density and (b) Ra and photosynthesis. Solid lines represent fitted regression models. Ra was calculated as Rs minus Rh.

Table S1. Summary of simple linear regression results for the relationships between plant activity indicators and respiration components. The table presents the slope (β), *p*-value, 95% confidence interval, adjusted R^2, and RMSE for the relationships of sap flux density and photosynthesis with Rs, Rh, and Ra.

Plant activity indicator	Respiration component	n	β (Slope)	*p*-value	95% CI	Adjusted R^2	RMSE
Sap flux density	Rs	178	108.84	<0.001	[88.41, 129.27]	0.42	283.77
Photosynthesis	Rs	178	85.26	<0.001	[68.94, 101.58]	0.37	294.19
Sap flux density	Rh	159	53.74	<0.001	[35.18, 72.30]	0.19	232.02
Photosynthesis	Rh	159	30.55	<0.001	[18.22, 42.89]	0.13	241.24
Sap flux density	Ra	146	24.83	<0.001	[17.82, 31.84]	0.36	78.42
Photosynthesis	Ra	146	19.03	<0.001	[13.99, 24.07]	0.29	77.47

Table S2. Peak lag times and cross correlation results between plant activity indicators (sap flux density and photosynthesis) and soil respiration components (Rs, Rh, and Ra). Peak lag is the lag time with the maximum cross correlation, and *r* is the corresponding correlation coefficient. n is the number of paired observations used after excluding missing values and outliers. Significance was evaluated using a block permutation test, and Pearson *p* values are also reported. NA indicates no significant lagged correlation.

Plant activity indicator	Respiration component	n	Peak lag (h)	*r* at peak	Pearson *p*	Block permutation *p*
Sap flux density	Rs	3646	13	0.31	$p < 0.05$	$p < 0.001$
Photosynthesis	Rs	4453	13	0.23	$p < 0.05$	$p < 0.05$
Sap flux density	Rh	3465	NA	-0.05	$p > 0.05$	$p > 0.05$
Photosynthesis	Rh	4132	NA	-0.08	$p > 0.05$	$p > 0.05$
Sap flux density	Ra	3465	13	0.32	$p < 0.05$	$p < 0.001$
Photosynthesis	Ra	4139	13	0.30	$p < 0.05$	$p < 0.001$
